# Gene discovery and transcript analyses in the corn smut pathogen *Ustilago maydis*: expressed sequence tag and genome sequence comparison

**DOI:** 10.1186/1471-2164-8-334

**Published:** 2007-09-24

**Authors:** Eric CH Ho, Matt J Cahill, Barry J Saville

**Affiliations:** 1 Department of Medical Biophysics, University of Toronto; Program in Genetics and Genomic Biology, The Hospital for Sick Children Research Institute, TMDT Building 14th Floor East Tower, 101 College Street, Toronto, ON, M5G 1L7, Canada; 2 Department of Genetics, University of Cambridge, Downing Street, Cambridge, CB2 3EH, UK; 3 Forensic Science Program, Trent University, DNA Building, 1540 East Bank Drive, Peterborough, ON, K9J 7B8, Canada

## Abstract

**Background:**

*Ustilago maydis *is the basidiomycete fungus responsible for common smut of corn and is a model organism for the study of fungal phytopathogenesis. To aid in the annotation of the genome sequence of this organism, several expressed sequence tag (EST) libraries were generated from a variety of *U. maydis *cell types. In addition to utility in the context of gene identification and structure annotation, the ESTs were analyzed to identify differentially abundant transcripts and to detect evidence of alternative splicing and anti-sense transcription.

**Results:**

Four cDNA libraries were constructed using RNA isolated from *U. maydis *diploid teliospores (*U. maydis *strains 518 × 521) and haploid cells of strain 521 grown under nutrient rich, carbon starved, and nitrogen starved conditions. Using the genome sequence as a scaffold, the 15,901 ESTs were assembled into 6,101 contiguous expressed sequences (contigs); among these, 5,482 corresponded to predicted genes in the MUMDB (MIPS *Ustilago maydis *database), while 619 aligned to regions of the genome not yet designated as genes in MUMDB. A comparison of EST abundance identified numerous genes that may be regulated in a cell type or starvation-specific manner. The transcriptional response to nitrogen starvation was assessed using RT-qPCR. The results of this suggest that there may be cross-talk between the nitrogen and carbon signalling pathways in *U. maydis*. Bioinformatic analysis identified numerous examples of alternative splicing and anti-sense transcription. While intron retention was the predominant form of alternative splicing in *U. maydis*, other varieties were also evident (e.g. exon skipping). Selected instances of both alternative splicing and anti-sense transcription were independently confirmed using RT-PCR.

**Conclusion:**

Through this work: 1) substantial sequence information has been provided for *U. maydis *genome annotation; 2) new genes were identified through the discovery of 619 contigs that had previously escaped annotation; 3) evidence is provided that suggests the regulation of nitrogen metabolism in *U. maydis *differs from that of other model fungi, and 4) Alternative splicing and anti-sense transcription were identified in *U. maydis *and, amid similar observations in other basidiomycetes, this suggests these phenomena may be widespread in this group of fungi. These advances emphasize the importance of EST analysis in genome annotation.

## Background

*Ustilago maydis *is a ubiquitous pathogen of *Zea mays *(corn) that can cause tremendous economic losses [1]. The most conspicuous symptom of the smut disease *U. maydis *causes is a fungus-induced tumour containing dark diploid teliospores. The spores disperse and germinate to produce saprophytic haploid cells. Compatible haploids fuse to form the filamentous dikaryon that penetrates the plant initiating a new round of infection.

*U. maydis *has been developed as a model for fungal plant pathogenesis because it is readily cultured in the laboratory and is amenable to genetic analysis and molecular manipulation. A draft sequence of the 20.5 Mb genome was released in 2004 [2] and a physical map of the 23 chromosomes was assembled using bacterial artificial chromosome clones [3]. Full utilization of the genome sequence requires its thorough annotation. Critical to this annotation are the determination of transcript sequences, and an indication of when transcripts are expressed. The work described here produced cDNA sequence information for genome annotation and was carried out in the context of investigating *U. maydis *response to nutrient limitation and aspects of *U. maydis *transcript structure that provide insight into the control of gene expression. The data obtained will strengthen the model and may provide insight that allows us to combat fungal pathogens in the field.

A critical aspect of pathogenic development by *U. maydis *is sensing and responding to nutrient availability and other signals from the host [4,5]; yet the mechanism of control is not known. A widely conserved aspect of nitrogen metabolism in fungi is nitrogen catabolite repression (NCR, [6]). During NCR in fungal species such as *Saccharomyces cerevisiae, Neurospora crassa *and *Aspergillus nidulans*, the presence of a preferential nitrogen source such as ammonia acts to suppress expression of enzymes that utilize other, less preferred, nitrogen sources [6-9]. During de-repression, the genes involved in metabolizing alternate nitrogen sources were induced when the preferred nitrogen source is absent and a specific alternate source is present [6]. The expression pattern results from a two step control mechanism coordinated by both global (e.g. the GATA binding *NIT2 *in *N. crassa *and *AREA *in *A. nidulans*) and pathway-specific transcription factors (e.g. *NIT4 *in *N. crassa*, [6]). In the rice blast pathogen *Magnaporthe grisea *the NIT2/AREA-like master regulator is dispensable for pathogenesis but is required for full expression of the pathogenesis gene *MPG1 *[10,11] and deletion of a GATA transcription factor in the bean pathogen *Colletotrichum lindemuthianum *severely reduces its pathogenesis [12]. Further evidence for the link between nitrogen metabolism and pathogenesis has come via genetic analysis of *M. grisea *that identified two genes that were regulators of NCR and pathogenesis [11]. Microarray hybridization experiments indicated *M. grisea *genes expressed during nitrogen limitation were also expressed during *in planta *growth [13,14]. Together, these discoveries suggest that, like other plant pathogens, control of nitrogen metabolism in *U. maydis *may have implications for pathogenesis, thus analysis of gene expression in nitrogen limiting conditions is relevant to pathogenesis as well as genome annotation.

As a further aid to genome annotation, analysis of transcript sequences also identifies introns and variation in intron splicing. Alternative pre-mRNA splicing is recognized as a significant contributor to proteome diversity in "higher" metazoans, with an estimated 74 % of human genes giving rise to alternately spliced transcripts [15]. In contrast, among the fungi, only *M. grisea *and *Cryptococcus neoformans *have been subject to genome-scale assessment of alternative pre-mRNA splicing [16,17]. The examination of expressed sequence tag (EST) libraries from several *M. grisea *strains identified 134 instances of alternative splicing among 8,177 unique expressed sequences (1.6 %). In this *M. grisea *analysis, intron retention accounted for the vast majority of the alternate splicing events. The annotation of the *C. neoformans *genome sequence [17], noted 277 examples of alternative splicing among 6,594, predicted gene models (4.2 %). Among the alternative splicing events noted for *C. neoformans*, were numerous examples of exon skipping and alternative splice site selection. Investigating what types of alternate splicing occur in *U. maydis *will provide information for genome annotation and for assessing the diversity of alternate splicing in the fungi.

There is some knowledge of the diversity in expression of antisense RNAs among fungi. In *Schizosaccharomyces pombe*, small antisense transcripts are produced through a conserved mechanism and function in heterochromatin organization and cell division [18,19] and in *N. crassa *small antisense RNAs are involved in quelling and classic RNA inhibition (RNAi) [20,21]. However, *S. cerevisiae *lacks the main components of the RNAi machinery [22] and no classic examples of RNAi have been reported for this fungus. In contrast, there is evidence of a non-classical gene suppression mechanism involving RNAs complementary to the 5' untranslated region (UTR) in *S. cerevisiae *[23] Therefore, the types of small antisense transcripts that are involved in controlling gene expression during transcription, splicing, mRNA degradation, gene silencing and imprinting in an evolutionarily broad range of organisms (for reviews see [24-26]) are not present in all fungi. This suggests that, for fungi lacking the classic RNAi machinery, there may be other mechanisms for controlling these processes. Comparative genomics analyses showed that *U. maydis *also lacks the main components of the RNAi machinery [27]. Furthermore, antisense RNA was shown to be unable to suppress gene expression in *U. maydis *[28]. However, the presence of natural antisense transcripts has not been investigated. The identification of antisense RNAs in *U. maydis *would aid genome annotation and may provide insight into mechanism of controlling gene expression not described for other eukaryotes.

Previous *U. maydis *EST libraries [29,30] were utilized in the original automated annotation of the draft genome sequence by the Broad institute [2]. The goals of the current work were three fold: 1) to aid in thorough genome annotation [31], 2) to determine differences in transcript abundance of genes expressed under nitrogen and carbon starvation and 3) to conduct structural analyses of transcripts aimed at the identification of alternate splicing and antisense transcription in *U. maydis*.

## Results

### Morphological and growth rate changes associated with nutrient limitation

**Figure 1:**
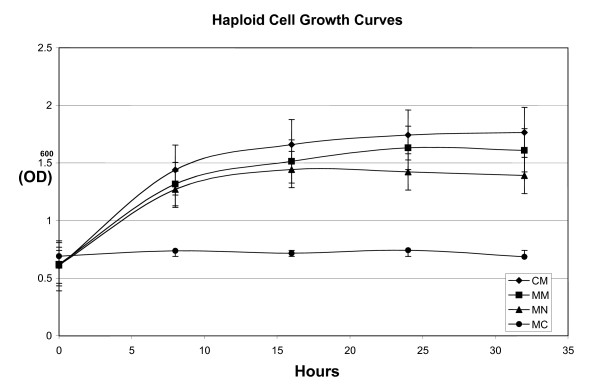
Growth curves for *U. maydis *growing under nutrient limitation: growth curves and photomicrographs. Growth curves of haploid cultures of *U. maydis *grown in complete media (CM), minimal media (MM), carbon starvation media (MC) and nitrogen starvation media (MN). Each point on the graph is an average of three biological replicates, standard error bars were included.

Examination of nutrient starved *U. maydis *haploid cells showed a distinct difference in the response to nitrogen and carbon limitation (Figure 1). The cells appear to actively adapt allowing for growth and division in the absence of exogenous nitrogen. When the haploids were transferred from CM to minus nitrogen media (MN), the rate of growth, in each of three replicates, decreased and reached a stationary phase earlier than haploids maintained in complete media (CM) or minimal media (MM; Figure 1). The cells grown in minus nitrogen media were also highly vacuolated relative to those growing in CM, MM or minus carbon media (MC) suggesting that changes in cytology as well as physiology were part of this adaptation (Data not shown). In contrast, the haploids grown in MC appear to stop mitotic division and were not distinct from cells grown in CM or MM (Data not shown).

### Sequences analysis, contig assembly and gene assignment

Four cDNA libraries were constructed using total RNA from *U. maydis *dormant teliospores (TD), pooled from several infected corn plants, and mRNA from haploid cells grown in CM, MN and MC. A total of 20,712 sequencing reactions were performed, the TD, MN and CM libraries contributed 4,992 reactions each and the MC library the remaining 5,736 reactions. After quality assessment, a total of 15,901 ESTs were generated with an average size of 473 bp. The BLASTN algorithm was used to search the ESTs against the *Ustilago maydis *genome sequence assembly [2], 15,848 ESTs produced a significant alignment (Table 1).

**Table 1:** cDNA/EST Library Statistics

	**CM**	**MC**	**MN**	**TD**	**Total**
**Sequence Reactions**	4992	5736	4992	4992	20712
**Number of Quality Sequences (QS)**	4043	4490	4133	3235	15901
**Average Size of QS (bp)**	500	484	456	446	N/A
**Predicted Full Length ESTs**	2460	3368	1152	1827	8807
**Number of Contigs**	1546	1120	1981	1611	N/A
**Number of Exclusive Contigs**	415	237	816	673	2141
**Redundancy (EST/Contig)**	2.6	4.0	2.1	2.0	N/A
**EST with Genome Hit**	4019	4490	4104	3235	15848

The ESTs that aligned to the genome were assembled into 6,101 contigs based on genome location. The average genomic length of a contig was 641 bp. ESTs that overlapped or were adjacent to each other were combined into a contig. The accuracy of contig assembly was increased by the addition of 9,014 ESTs from the germinating teliospores and the filamentous diploid libraries [29,30]. Based on the mean number of ESTs/contig the TD and MN libraries were the least redundant (Table 1).

A predicted gene set that was compiled by The Munich Information Center for Protein Sequences (MIPS) consists of the Broad Institute's autocalled gene set, MIPS own predicted genes, and formerly identified genes. The analyses herein were based on the February 2006 version of the gene set. Assembled contigs were assigned a predicted gene identity based on genome location as described in the Methods. Of the 6,101 contigs assembled, 5,482 contigs were assigned to 4,274 unique MUMDB genes. The 619 remaining contigs aligned to the genome sequence but not to regions corresponding to MUMDB genes; 218 out of the 619 contigs were anti-sense (described below). Only 63 of the remaining 401 contigs produced a significant alignment when searched against the NCBI non-redundant database using the BLASTX algorithm. Of the 63 matches, 58 were to old *U. maydis *predicted genes that have now been either discarded or re-model by MIPS manual annotation. The remaining five aligned to hypothetical, putative, or conserved hypothetical proteins of other fungi.

**Figure 2:**
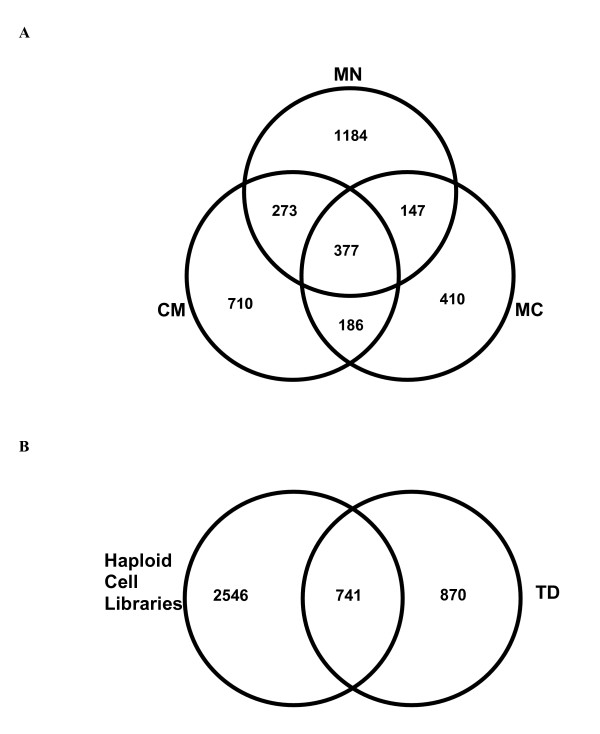
Distribution of Contigs among Libraries: Venn Analysis. Venn distribution of contigs a) among the haploid libraries, and b) between the haploid libraries and the dormant teliospores library.

The new libraries (MC, MN, CM, and TD) contribute a total of 4,157 contigs of which 2,566 (42 %) were not represented in published libraries. Among the new libraries, those constructed using haploid cells (CM, MC, MN) contributed 2,546 contigs while TD contributes 870 contigs and 741 contigs were common to TD and the haploid libraries (Figure 2). Distribution of the contigs within the haploid cell libraries indicates that MN has the most contigs present only in this growth condition and absent from others (1,184 contigs) while MC has the least (710 contigs). There were 150 contigs representing transcripts found in all 6 libraries (Additional File 1) that could represent members of a core gene set for *U. maydis*. A core gene set is a group of genes that are absolutely required for an organism to complete its life cycle. About a third of these contigs were ribosomal proteins. The 150 contigs were searched against the *S. cerevisiae *gene set [32], 116 contigs aligned and 27 of these to essential genes. Furthermore, there were 13 contigs whose functions were assigned as conserved hypothetical or hypothetical proteins, 4 putative proteins and four contigs that were not assigned a function. Contigs that did not align to *S. cerevisiae *genes were also searched against the non-redundant database of NCBI, using the BLASTX algorithm and no matches were found; suggesting these 21 contigs may represent taxon specific genes.

### Functional characterization

The contigs were grouped into functional categories based on the FUNCAT system proposed by MIPS [33]. At the time of this analysis, 2,420 of the 6,902 MUMDB genes had a FUNCAT assignment. Correspondingly, 1872 functionally categorized genes were represented in this work by 2,506 contigs. The distribution of the FUNCAT categories for the haploid libraries and the TD library, based on contig and EST numbers, were determined. The percentage of expressed genes assigned to functional categories was similar for all the libraries; however, the exact composition of the genes in a category varies (Additional File 2). Furthermore, the differences in the number of ESTs per category suggest that transcript abundance within a category varies among the four libraries (data not shown).

### Differential expression

To screen for contigs for which there were differentially abundant transcripts, *R *statistics [34] were calculated based on EST representation in the four libraries. A list of 25 contigs with the highest *R *statistical values was generated for further analysis (Table 2). There were eight contigs that do not have a known function, six were MUMDB proteins and two were not similar to characterized proteins (one corresponds to the highest *R *statistical value). The last two may represent *U. maydis*-specific genes. Seven contigs where comprised entirely of ESTs from the dormant teliospores library. Eight contigs (Contig 1438, 4572, 4070, 1652, 2167, 3235, 1015, 3380) were chosen for reverse transcriptase quantitative PCR (RT-qPCR) to investigate the differential transcript abundance predicted by *R *statistics analysis (Table 2). RT-qPCR reactions were performed on RNA isolated from haploid cells grown under different nutritional conditions along with RNA from the dormant teliospores. Results confirm the differential transcript abundance predicted by the *R *statistics in the instances where the libraries compared were constructed by the same method (contigs 4572, 1652, 2167, 1015 and 3380, (Additional File 3).

**Table 2:** Differentially regulated genes as predicted by *R *statistical analysis

**CONTIG ID**	**CM**	**MC**	**MN**	**TD**	**R-Values**	**MIPS ID**	**Description**
CONTIG 879	41	392	87	34	97.49	--	unknown
CONTIG 5036	0	0	0	92	63.44	um01778	putative protein
CONTIG 1030	0	0	0	72	49.65	um03881	putative protein
**CONTIG 1438**	**13**	**134**	**6**	**8**	**46.39**	**--**	**unknown**
CONTIG 1199	146	54	16	8	41.13	um11054	probable FPR1 – peptidyl-prolyl cis-trans isomerase, FK506-binding protein
**CONTIG 4572**	**0**	**0**	**0**	**59**	**40.68**	**um01426**	**conserved hypothetical protein**
CONTIG 337	25	123	5	5	40.36	um00496	Mismatch base pair and cruciform DNA recognition protein Hmp1
CONTIG 4303	2	82	1	6	34.83	um10956	probable RPP1A – 60S large subunit acidic ribosomal protein a1
CONTIG 2911	5	16	1	73	33.58	um00205	related to HSP12 – heat shock protein
*CONTIG 3235	**0**	**0**	**47**	**0**	**27.54**	**um05889**	**High Affinity Ammonium transporter**
CONTIG 5954	10	75	2	6	25.24	um10114	probable 40S ribosomal protein S16
CONTIG 1263	0	0	0	36	24.82	um04125	probable YRO2 – strong similarity to HSP30 heat shock protein Yro1p
**CONTIG 4070**	**21**	**86**	**20**	**2**	**21.28**	**um00919**	**probable ADP, ATP carrier protein (ADP/ATP translocase)**
**CONTIG 1652**	**0**	**0**	**0**	**27**	**18.62**	**um04481**	**related to Alcohol dehydrogenase**
CONTIG 2437	1	0	6	33	17.57	um05243	hypothetical protein
**CONTIG 2167**	**7**	**0**	**4**	**35**	**16.53**	**um04974**	**putative protein**
CONTIG 1840	6	40	0	2	15.55	um11551	probable RPS19B – ribosomal protein S19.e, cytosolic
CONTIG 3425	0	29	2	0	13.86	um06055	probable RPP0 – acidic ribosomal protein L10.e
CONTIG 3229	36	20	2	1	12.46	um05880	probable ERG25 – C-4 methyl sterol oxidase
CONTIG 5978	33	44	9	1	12.04	um02708	probable CYTOCHROME C
CONTIG 2481	0	0	0	17	11.72	um05264	probable glutathione-dependent formaldehyde dehydrogenase
*CONTIG 1015	**0**	**0**	**19**	**0**	**11.13**	**um11105**	**related to nitrate transporter**
CONTIG 2106	3	27	1	0	11.03	um04922	related to 2,5-diketo-D-gluconic acid reductase
*CONTIG 3380	**0**	**0**	**20**	**1**	**10.66**	**um06012**	**probable general amino acid permease**
CONTIG 177	0	0	0	15	10.34	um03050	hypothetical protein
CONTIG 4050	48	13	29	4	10.02	um00924	probable translation elongation factor eEF-1 alpha chain

List of contigs representing genes identified as differentially regulated. MIPS gene functions were also included. Genes investigated by RT-qPCR were in bold. Genes denoted by an asterisk were involved in nitrogen metabolism.

**Figure 3:**
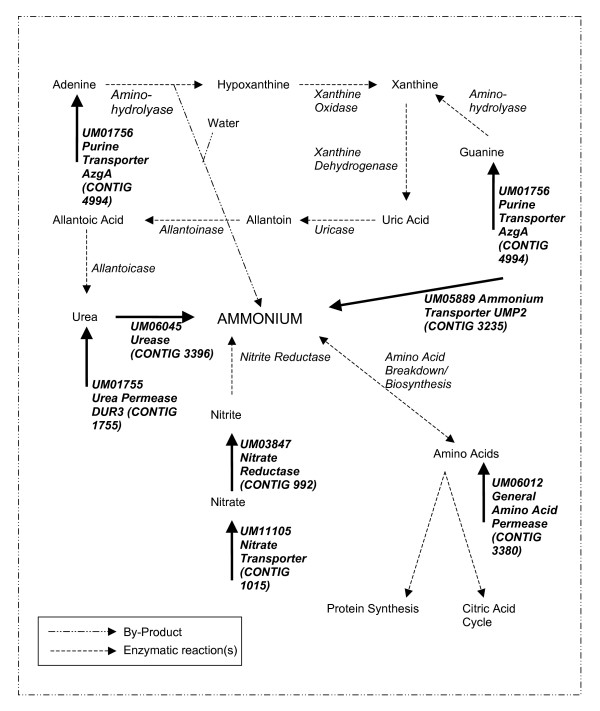
Fungal nitrogen metabolism pathways. The metabolic context of the proteins, for which transcript abundance was assessed by RT-qPCR, is shown. The proteins assessed are indicated by bold font. These include purine transporter (E.C. 2.A.39.2.1), ammonium transporter (E.C. 2.A.49), urease (E.C.3.5.1.5.), urea permease, nitrate reductase (1.7.1.2), nitrate transporter (E.C. 3.6.3.26) and general amino acid permease.

Differential transcript abundance of genes involved in nitrogen metabolism was also investigated by RT-qPCR. Three of the eight contigs that were examined for confirmation of the *R *statistic predictive value were up regulated in the nitrogen starved library (Contig 3380: probable general amino acid permease, 1015: nitrate transporter, and 3235: High affinity ammonium transporter UMP2). The transcript abundance of these genes, as well as that of four other nitrogen metabolism genes represented in the libraries (bold in Figure 3), was investigated in 3 biological replicates of the haploid cells and teliospores pooled from different infected plants following two independent series of inoculations. During this investigation haploid cells grown in minimal media (MM) were included as a control since MN and MC were derivatives of MM. The RT-qPCR results displayed the pattern of transcript abundance predicted from the *R *statistic analysis (Additional File 3). In addition, for all nitrogen metabolism genes investigated, the results showed that the abundance of transcript was similar in MN and MM but reduced in MC. Results that suggest carbon availability has a role in controlling nitrogen metabolism gene transcript abundance since MM and MC had the same nitrogen source and concentration but differed in carbon source availability.

### Transcript structure generation

The alignment of 24,643 5' ESTs to their cognate genomic loci resulted in the identification of 6,284 predicted gene locations or PGLs. Alignments carried out in this manner provided an independent prediction of potential gene locations and not all PGLs corresponded to the MUMDB ORFs. On average, each PGL was represented by 3.9 ESTs; although, the majority (3,463 PGLs) were represented by only a single EST. Positional assignment of the PGLs to MUMDB genes showed that 5,484 (87 %) can be unambiguously paired to 4,307 MUMDB genes, while 800 (13 %) do not correspond to a predicted gene. As such, a definitive estimate of the proportion of *U. maydis *genes represented by the 6284 PGLs was not possible; however, it is at least 62 % (4,307/6,902) of the genes in MUMDB.

**Figure 4:**
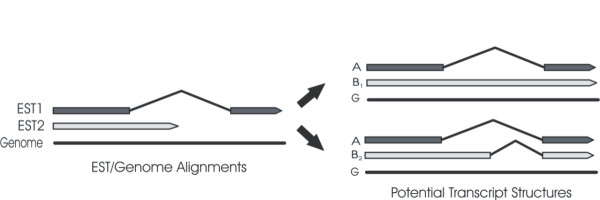
Impact of 5-prime limited ESTs on the categorization of AS events. In this representative example, the alignment of EST1 to the genome sequence identifies an intron in the cognate gene. EST2 reads into this intron and thus provides evidence of the alternative splicing of this gene. However, as EST2 does not extend through the entire intron, the sequence data supports either retention of the entire intron (B_1_), or that there was an alternative donor site (B_2_).

Note that the majority of the gene structures generated by GeneSeqer were expected to be partial; reflecting the 5' limitation of the ESTs upon which they were based (Figure 4). Where relevant, the implications of this bias will be addressed.

### Identification of alternative splicing events

GeneSeqer assigned single gene structures to 6,004 of the 6,284 PGLs that were initially identified. The remaining 280 PGLs were assigned at least 2 structures; an observation that suggests multiple mRNA isoforms originate from these loci. Direct examination using MyGV 1.0 [35] resulted in the identification of 224 PGLs for which strict EST/genome alignments supported the existence of more than one mRNA isoform. The assignment of more than one gene structure to a given PGL was taken as preliminary evidence of the alternative splicing of these loci.

The number of gene structures assigned to each of the 224 alternatively spliced PGLs ranged from 2 – 5. The 224 PGLs represent 3.6 % of the 6,284 identified by Geneseqer. However, it should be noted that only 874 of the original PGLs contained at least one intron and were supported by multiple ESTs: characteristics that were required for the detection of alternative splicing. Of this subset, 26 % had evidence of alternative splicing. The PGLs with evidence of alternative splicing as well as the corresponding MUMDB genes were presented in Additional File 4.

### Categorization of alternative splicing events

Having detected alternate splicing in *U. maydis *the structures predicted for each PGL were further analysed to determine, where possible, the nature of each alternate splicing event. Two issues became evident during this analysis: 1) The 5-prime limitation of the ESTs often meant that the nature of alternative splicing events may be ambiguous (e.g. see Figure 4), and 2) There was difficulty in distinguishing between alternative splicing and multiple transcriptional start sites in some cases. In both instances, the PGL in question was considered alternatively spliced, but was not categorized (Additional File 4). Among the 224 PGLs, there was sufficient information to unambiguously categorize 142. Examples of all varieties of alternative splicing were present; however, the majority consisted of intron retention events.

ESTs containing "retained" introns may arise by contamination of the RNA starting material with genomic DNA (gDNA) or the presence of unprocessed transcripts. Contaminating gDNA was eliminated by selective RNA isolation and DNase treatment (See Methods). The efficacy of these measures was assessed, prior to library construction, using RT-PCR amplification directed at the detection of unspliced transcripts of the *U. maydis *glycogen synthase kinase gene (*ugk*, Data not shown). Under all nutrient conditions and for all cell types, no gDNA contamination was detected. Therefore, gDNA contamination cannot account for those ESTs containing retained introns. The RT-PCR screen also indicated that no unprocessed *ugk *transcripts were present in the mRNAs used for library construction; confirming that the intron retention identified was legitimate and not a transcript captured before splicing had occurred. However, as different genes may have different rates of mRNA processing the *ugk *screen may not reflect the state of all transcripts represented in the library and it was conceivable that some proportion of the ESTs may be derived from unprocessed pre-mRNAs. As such, all transcript structures containing retained introns were subcategorized to reflect the relative confidence that the intron was retained in a mature mRNA (as opposed to a pre-mRNA). The categories were as follows: Single EST IR – Intron retention was implied by only a single EST, Multiple EST IR – Intron retention was implied by multiple ESTs, and Processed IR – An intron was retained in a transcript structure that also showed successful splicing of another intron. A summary of the categorization is presented in Table 3.

**Table 3:** Categories of Alternate Splicing Detected

**Category**	**Occurrences**
Intron Retention – Single EST	38
Intron Retention – Multiple ESTs	32
Intron Retention – Processed	44
**SubTotal **	**114**
Alternate Donor Site	23
Alternate Acceptor Site	44
Exon Skipping	12
**Total^1^**	**193**

While the number of alternatively spliced PGLs that could be unambiguously characterized was 142, many of these were subject to more than one splicing event and, in this tabulation, each splicing event was counted.

A comparison of the *U. maydis *genes for which there is evidence of alternate splicing to the genes identified as being alternatively spliced in *C. neoformans*, indicates nine genes that are similar (> e-20). The *U. maydis *(and corresponding *C. neoformans *genes) are: um00945 (XP_571553.1 and XP_571554.1), um01221 (XP_568869.1 and XP_568870.1), um02412/10149 (XP_571981.1 and XP_571982.1), um05644 (XP_568635.1 and XP_568636.1), um10221 (XP_566828.1 and XP_566829.1), um10238 (XP_570671.1 and XP_570672.1), um11098 (XP_566793.1 and XP_566794.1), um11210/um06111 (XP_569233.1 and XP_569234.1), and um11769 (XP_567037.1 and XP_567038.1). One of these genes, isocitrate dehydrogenase (um11210/um06111) has also been identified as being alternatively spliced by MUMDB. um11210 is predicted to be the cytosolic enzyme and um06111 the alternately spliced mitochondrial form. Isocitrate dehydrogenase has also been shown to be alternately spliced in humans [36]. In both humans and *U. maydis *the result of alternate splicing is an alteration in the N terminal exon of the gene. More data is required to determine the similarity in splice junctions.

In order to provide some evidence in support of alternate splicing apart from the EST analysis RT-PCR amplifications were carried out from two biological replicates of each cell type. The results are presented in Additional File 5 and a diagrammatic representation of the predicted alternate splicing events in Additional File 5. Note that Additional File 5 only represents the portion of the transcript affected by the alternate splicing and does not reflect transcript start or stop sites. In Additional File 5 separate gels are aligned so that separate amplifications from the same template cDNA appear in the same lane. Primers were selected to amplify regions that would vary in size if the alternate gene structures or AGSs predicted in silico were present as transcripts. Four transcript structures were predicted for um04632 (top of Additional File 5). The structures are the results of alternative acceptor selection (structure 1 in Additional File 5), intron excision (structure 2), retention of a single intron (structure 3), and retention of two introns (structure 4). The predicted amplicons for the four AGSs of um04632 were 1) 347 bp, 2) 444 bp, 3) 207 bp, and 4) 987 bp. Amplification of the genomic DNA would yield a 987 bp fragment. Structure 2 had the most EST support and an amplicon corresponding in size to that predicted by this structure is the brightest stained band in all lanes except in the amplification from genomic DNA (Additional File 5). A band corresponding in size to that predicted for structure 1 is visible in the CM, MN, TD and DIP lanes where as a band corresponding to structure 4 is visible in the MC lane. No band corresponding to structure 3 was detected; however, this structure was predicted based on ESTs from the germinating teliospores library constructed previously and this condition was not included in the RT-PCR experiment. A band, of approximately 900 bp, not predicted from the ESTs analyzed for um04632, was present in the Dip lanes. For the gene um10506/7 two AGSs were predicted, the structures illustrating this are shown in Additional File 5, structure 2 had the greater EST support, it would yield an amplicon of 490 bp and structure 1 would yield an amplicon of 319 bp. Both amplicons are present in all lanes except the gD lane. Faint bands of slower mobility than that corresponding to the AGS1 amplicon are also present in some lanes. No ESTs consistent with these amplicons were analyzed. For um11744 three structures were predicted, these would be represented by amplicons of 360 bp, 396 bp and 514 bp respectively. The first two amplicons were not resolved on the gel picture included. For the gene um02514 two AGSs were predicted and DNA amplicons consistent in size with these were detected, amplicons were, for structure 1 – 257 bp and structure 2 – 79 bp. Finally the control um 00560, *ugk*, has been confirmed to have a single intron which is constituently spliced (Saville in preparation) and amplicons produced are consistent in size with the excision of this intron. In summary the results show the presence of multiple transcript isoforms, the majority of which are consistent with the alternately spliced mRNAs predicted by EST analysis, and provided evidence for the cell type or nutrient response specific production of mRNA species.

### Characteristics of *U. maydis *introns

In addition to detecting PGLs with evidence of alternative splicing, the gene structures generated by GeneSeqer identified EST-supported introns. While there exists a manually refined, software generated, collection of *U. maydis *gene models, this analysis was restricted to those introns supported by ESTs. The restriction to EST supported introns allowed the identification of those subject to alternative splicing to be distinguished from those that, based on available evidence, were constitutively spliced.

The 280 PGLs for which there is preliminary evidence of alternative splicing were separated from the original 6284 prior to the assembly and analysis of the intron dataset. Subsequently constitutively spliced introns were compared with those subject to alternative splicing. Among the remaining 6,004 PGLs, 1,146 introns were identified in 821 PGLs. The number of introns per PGL ranged from 1 to 6, though the majority 584 contained only a single intron. Of the 1,146 introns identified, 6 introns were discarded based on length and 58 did not bear the accepted splice site dinucleotides (see methods). The EST/genome alignments supporting these 58 introns were examined (using MyGV); this showed that 29 were the result of mis-alignment and/or sequencing errors while the remaining 29 appeared to be genuine introns. Thus, the filtered dataset consisted of 1111 introns, 29 that bore non-canonical splice site dinucleotides.

The presence of 1,111 introns among the 6,004 PGLs corresponded to a frequency of 0.19 introns/PGL. While the 5' limitation of the ESTs precludes any interpretation of this frequency on a "per gene" basis, the observed frequency does not differ markedly from the 0.48 that was arrived at using manually curated gene models [31]. The EST supported introns ranged in size from 24 – 1,899 nts, with a mean of 169.8 nts. A frequency histogram of the intron lengths is presented in Additional File 6.

**Figure 5:**
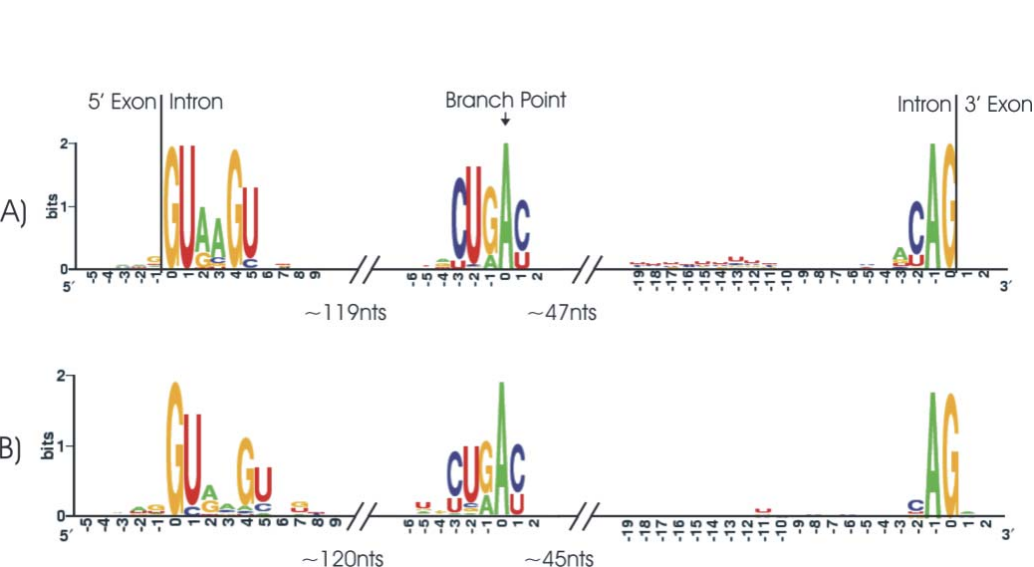
Sequence logos of *U. maydis *intron splice sites and branch point. a) Logos of constitutively spliced introns. b) Logos of retained introns.

Putative branch sites could be identified in 1,079 of the 1,082 introns that passed filtering and bore canonical splice sites. Sequence logos based on the alignments of the Intron/Exon borders and the putative branch points were presented in Figure 5a.

### Characteristics of retained introns

A collection of 49, well supported (Processed IR), retained introns was analyzed using the same methodology as that employed in the analysis of constitutively spliced introns. The retained introns ranged in length from 30 – 743 with a mean of 148 nts. None of the introns were removed from the analysis based on size but 18 (38 %) did not contain acceptable splice site dinucleotides. The EST/Genome alignments supporting these introns were examined and none were found to be the result of alignment or sequencing errors. Putative branch points could be identified in 29 of the 31 introns that bore canonical splice sites. Sequence logos based on the alignments of the splice sites and branch point were presented in Figure 5b. Comparison of Figures 5a and 5b indicate that the sequence features of the retained introns were more degenerate than those that were constitutively spliced.

### Anti sense transcript analysis

**Figure 6:**
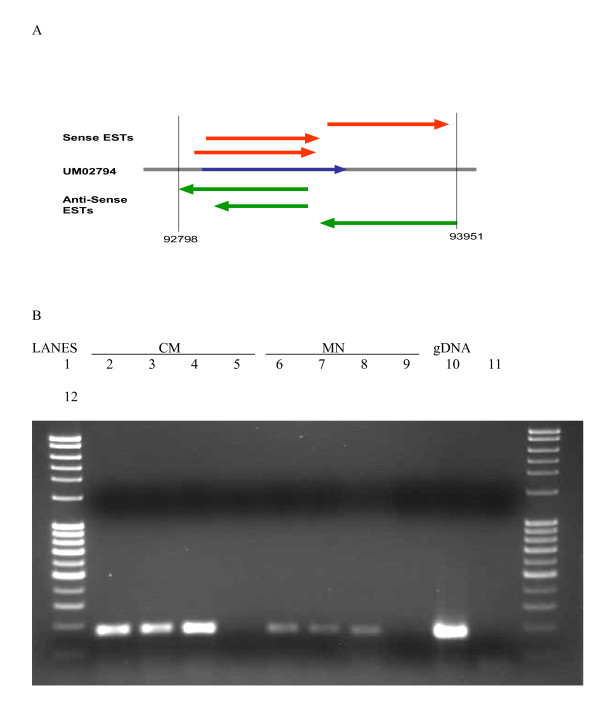
RT-PCR confirmation of antisense transcription. a) A graphic representation of um02794 transcription. The grey line represents the genomic sequence (middle), the blue arrow represents predicted gene structure, and the red (top) and green (bottom) arrows represent sense and anti-sense ESTs respectively. The range of the genome coordinates was included. b) Detecting antisense transcripts corresponding to um02794 via strand specific RT-PCR. In lanes 2 to 5 first strand synthesis was carried out on RNA of CM grown haploid cells. In lanes 6 to 9 first strand synthesis was carried out on RNA of MN grown haploid cells. First synthesis reactions of lanes 2 and 6 were prepared using sense strand specific primers; lanes 3 and 7 anti sense specific primers; lane 4 and 8 oligo dT and lanes 5 and 9 DEPC-treated water. Lane 10 used genomic DNA from *U. maydis *strain 521 and lane 11 used water a PCR template. Lane 1 and 12: Full Ranger DNA ladder.

Anti-sense transcripts were identified as contigs that aligned to the non-coding strand of a MUMDB gene. A total of 210 MUMDB genes, corresponding to 218 contigs, have anti-sense transcripts represented in the EST libraries. The presence of anti sense transcripts for 185 genes was supported by a single EST, where as 15 were supported by 2 ESTs and 10 were supported by 3 or more ESTs (Table 4). Many of the genes with antisense ESTs were also represented by sense ESTs in the libraries. (Table 4, Additional File 7). Manual analysis assisted by Spidey [37], was performed on seven genes represented by multiple anti sense and sense ESTs. One of the seven genes analyzed, um02794, was represented by three anti sense ESTs from the MN library and three sense ESTs from the CM library (Additional File 7). PCR was performed on either the sense or anti sense strand cDNA that was produced using strand specific primers. The RT-PCR results confirm the presence of that both anti-sense and sense transcripts in two independent pooled cultures of CM and MN grown haploid cells (Figure 6).

**Table 4:** Summary of antisense transcript occurrences in the *U. maydis *EST libraries

	Predicted Antisense Contigs/Regions	Predicted Antisense Contigs/Regions with"Sense" support
Single EST support	185	116
Double ESTs support	14	3
Multiple ESTs support	10	4

Genes were described fully in Additional File 5

## Discussion

### Growth and cellular response to nutrient starvation

When *U. maydis *haploid cultures were transferred from CM to MN media, the rate of growth decreased and the cells became vacuolated, a process that may be evolutionarily related to the autophagic response of *Candida albicans *and *S. cerevisiae *to nitrogen limitation [38,39]. In contrast to nitrogen starvation, the carbon starvation response appears to be marked by the cessation of cell division, but no obvious morphological changes (Figure 1). The visible responses of *U. maydis *to nutrient starvation were associated with changes in gene transcript abundance allowing us to use these responses to identify novel genes, advance the knowledge of transcript structure and contribute information to aid annotation of the genome.

### Contig assembly and functional annotation

The assembly of contigs based on the co-ordinates of an EST's genome location has two advantages over the approach used in earlier studies (e.g. [16,29,30,40-44], it removes the reliance on EST overlap, allowing accurate assembly of ESTs that align to adjacent sequences in the genome or share only a small overlap, and it eliminates the possibility that ESTs representing distinct genes will be assembled based on shared conserved functional domains. Knowledge of the genome location of the uniESTs also allows for assignment to predicted genes that, in turn, allows the utilization of existing annotation databases (e.g. FUNCAT). Contigs identified in this work correspond to 62 % (4,274) of the MUMDB ORFS (6902, [31]). Apart from these, 401 contigs corresponded to the genome but not to a MUMDB gene or an anti-sense strand variant, 58 of these corresponded to *U. maydis *genes that were predicted by previous annotation programs [2] but were rejected in more recent annotation models; this suggests that the current annotation requires continued updating and for this reason these data have been shared with MIPS. The remaining contigs did not have significant similarity to characterized protein or RNA coding genes suggesting they represent formerly unidentified *U. maydis *genes.

The ESTs representing transcripts not previously sequenced [29,30], were assembled into 2,565 contigs; a significant increase in genes represented by ESTs that was facilitated by the relatively small overlap between the libraries (Figure 2a and 2b). Forty three percent of the predicted genes represented in the libraries were assigned to functional categories using the FUNCAT system of MIPS [33], this compares to 35 % for the entire *U. maydis *gene set. The presence of distinct transcript profiles in separate libraries underlines the distinct cellular responses exhibited under different growth conditions and between haploid cells and diploid teliospores. The functional annotation provides insight into the nature of these differences.

The TD library was created from 'dormant' teliospores, and, as such, there was an expectation that transcript abundance would be low and that transcripts would be evenly distributed among the FUNCAT categories. However, the TD library has a relatively high percentage of ESTs in the metabolism, cell rescue/defence and virulence categories. For example, um04481, a gene related to ADH II an alcohol dehydrogenase (EC. 1.1.1.1), was found only in the TD library where it had large EST support (Table 2). The up-regulation was confirmed by RT-qPCR (Additional File 3). The high abundance of this and other transcripts may reflect Zahiri *et al*'s[45] suggestion that genes required for, or immediately after, teliospore germination were transcribed prior to dormancy and stored for future translation. Interestingly, among the genes for which there were differentially abundant transcripts according to *R *statistic analysis, half of the contigs representing genes of unknown function were composed of ESTs only from the TD library. While this might reflect the lack of knowledge about teliospore biology and gene expression, it also supports the idea that taxon specific genes are expressed in taxon specific structures [46] as teliospores are only produced by the smut and rust fungi.

The MC library has a relatively high EST representation in the protein synthesis category including a large contribution by probable ribosomal proteins. The presence of large amounts of ribosomal protein transcripts was somewhat perplexing, as the carbon-stressed haploid cells appeared to have stopped dividing, this might imply the stop of protein synthesis. In addition, protease or ubiquitin ESTs were not detected in the MC library, indicating the cells were not degrading proteins to feed new synthesis. The ribosomal protein transcripts might have been transcribed prior to the stop of cellular activities and then stabilized on the switch to carbon starvation conditions. In this state, the cells would be primed for ribosome production when a carbon source becomes available. In *S. cerevisiae*, short duration glucose withdrawal experiments, showed that translation was halted while mRNAs for ribosomal proteins remain intact [47]. In these, cells, translation recovered rapidly when glucose was re-introduced, and this recovery did not require new transcription [47]. In the *U. maydis *experiments, the period of carbon starvation was much longer and translation was not assessed; however, the existence of ribosomal protein transcripts suggests measuring translation before during and after carbon starvation may show that mRNAs were stabilized and not translated, a situation similar to the teliospore.

Morphological observations and growth curve determinations, suggested that *U. maydis *haploid cells grown in MN were rapidly adapting to the nitrogen stress environment. Consistent with this is the observed expression of genes to obtain and metabolize all available nitrogen sources. The increased transcript level for these genes suggested by *R *statistic analyses was confirmed by RT-qPCR.

### EST representation and differential expression

The frequency of the EST clones per contig across the different libraries was used to identify differentially abundant transcripts based on the R-statistical analysis of Stekel *et al*. [34]. RT-qPCR analysis of eight genes indicated that EST frequency in the MC library, constructed using the Cap-Trapper method, does not correspond to relative gene expression. The comparison here is between EST frequencies in libraries constructed by two different methods, the other libraries were constructed using a SMART cDNA kit. The results confirm those of previous studies that showed Cap-trapper and SMART cDNA library creation produce different distributions of ESTs [48]. They also support the contention that libraries constructed by different methods cannot be compared to provide information on the relative transcript abundance [34]. The statistical analysis does indicate, however, that the relative amounts of ESTs in the TD, CM and MN libraries reflect differences in transcript abundance. Furthermore, the RT-qPCR results provide insight into the *U. maydis *nutritional response.

### Metabolism of less favoured nitrogen sources

The seven *U. maydis *genes selected for RT-qPCR (Additional File 3) were from three different nitrogen metabolism pathways, each controlled by NCR in other fungi [6]. Previous investigation of *U. maydis *nitrate reductase *NAR1 *(um03847) showed that it was induced in the absence of ammonia and the presence of nitrate [49]. However, the expression of *NAR1 *in the absence of nitrate has not been assessed. RT-qPCR results showed that *NAR1 *was up-regulated in MN (no added nitrogen source) and in MM (nitrate as nitrogen source). While the level of nitrogen that may be leached from the cells transferred to the medium lacking a nitrogen source was not determined; it is clear that the cells under this low nitrogen condition were stressed. The low level of N is supported by the consistency of transcript level determinations with previous studies [50]. Under these conditions expression patterns similar to that of *NAR1*were found for the other nitrogen metabolism genes, suggesting that the control in *U. maydis *may be global. If this is the case then *U. maydis*, unlike most other fungi studied [6], may have a single step mechanism for regulating expression of genes for metabolism of alternate nitrogen sources.

In an attempt to determine how a single step mechanism might be controlled in *U. maydis*, genes similar in sequence to known regulators of nitrogen metabolism were considered in relation to characterized regulatory systems. MIPS predicted a gene similar to the pathway specific activator *NIT4 *(MUMDB annotation um02808) and BLAST searches carried out herein identified a *NIT2/AREA*-like global regulator um10417. In *A. nidulans *the alternate nitrogen metabolism genes acetamidase, histidase and formamidase are under the sole control of global activator *AREA *and do not require additional inducers for activation [8,51-53]; this could be the case in *U. maydis*. Another control mechanism is present in *Magnaporthe grisea *where genetic analysis indicates that two regulatory genes, *NPR1 *and *NPR2*, act upstream of a *AREA/NIT2-*like GATA transcription factor to maintain NCR and induce pathogenesis [11]. Like *U. maydis, M. grisea *alternate nitrogen metabolism genes were up regulated under nitrogen starvation without the presence of an alternate nitrogen source [14]. The similarities suggest that there may also be a global regulator or regulators in *U. maydis*.

The down regulation of nitrogen metabolism genes in MC (Additional File 3), even though MC and MM both contain nitrate as a nitrogen source, suggests that carbon starvation overrides the nitrogen metabolism regulatory system. In contrast, carbon starvation in *S. cerevisiae *induces the expression of some nitrogen metabolism genes. The cross talk between the carbon and nitrogen signalling pathways in *S. cerevisiae *occurs through *SNF1*, a protein kinase involved in carbon regulation that also interacts with *GLN3*, the global repressor of nitrogen metabolism, during carbon starvation [54-56]. The data here suggest that there was crosstalk between the nitrogen and carbon signalling pathways in *U. maydis *and that the outcome of this was a reduced quantity of transcripts for genes involved in nitrogen up take and metabolism.

### Characteristics of *U. maydis *introns

In addition to utility in gene discovery and the study of differential expression, sequenced cDNAs (in the form of ESTs) allow for the elucidation of gene structure; specifically, alignment of ESTs to corresponding genomic loci identifies introns within the genome sequence and may define transcriptional start and stop sites. Further, comparison of multiple cognate ESTs to one another may provide evidence of alternative pre-mRNA splicing. The software tool GeneSeqer was used to align the EST dataset to the *U. maydis *genome sequence, resulting in the identification of 6284 predicted gene locations. Of these, 280 were assigned more than one gene structure; an observation that was taken as preliminary evidence of alternative pre-mRNA splicing of these loci. In total, 1,141 introns were identified among the remaining 6,004 PGLs, a frequency of 0.19 introns/PGL which is somewhat less than the 0.48 introns/gene arrived at using manually-refined, software predicted, gene models [31]. The disagreement between this estimate and that of Kämper et al. [31] may result from the 5' limitation of ESTs analyzed here and the exclusion of PGLs subject to alternative splicing prior to estimation.

The intron dataset was analyzed using an approach largely identical to that, employed by Kupfer et al. [57], of introns in the ascomycete yeasts *S. cerevisiae *and *S. pombe*; the filamentous ascomycetes *A. nidulans *and *N. crassa*; and the pathogenic basidomycete *C. neoformans*. The vast majority of *U. maydis *introns contain the canonical 5'-GU....AG-3' splice site dinucleotides and have splice site and branch point sequences that were in general agreement with the introns of these other fungi (Figure 5). However, there were two noteworthy features of *U. maydis *introns, rarity, as discussed by Kämper et al. [31], and length. At 170 nts, *U. maydis *introns were larger than those of *C. neoformans *(69 nts), *A. nidulans *(73 nts), *N. crassa *(119 nts) and *S. pombe *(107 nts). Only *S. cerevisiae *(256 nts) has a greater average intron length and, like *U. maydis*, *S. cerevisiae *is intron-poor while the other fungi were comparatively intron-rich. Intron loss may be responsible for the predominance of larger introns in *U. maydis *and *S. cerevisae*. Intron loss could result from constraints on the splicing process leading to the preferential loss of small introns, or be the outcome of a stochastic process acting on an intron population that is dominated by shorter introns.

### Alternative splicing

Alternate splicing was predicted by in silico analysis and four genes were selected for RT-PCR amplifications aimed at confirming the existence of these predictions. The RT-PCR amplification of cDNAs not represented in the libraries suggested that post-transcriptional processing is more complex than predicted. However, the presence of DNA amplicons consistent in size with the EST analysis predictions (Additional File 5) suggested that alternately spliced transcripts are present in *U. maydis*.

There have been numerous reports of alternative pre-mRNA splicing in fungi [17]. However, the majority of these refer to single genes, or gene families. In contrast, the identification here of 224 instances of alternate splicing among 6,284 *U. maydis *PGLs analyzed, along with the confirmation by RT-PCR, constitute one of only a small number of transcriptome-level investigations of this process in a fungus. As part of the *C. neoformans *genome sequence annotation, Loftus et al. [17] identified 277 examples of alternative splicing among 6,594, predicted gene models (4.2 %). Ebbole et al.'s [16] analysis of *M. grisea *ESTs led to the discovery of 134 instances of alternative splicing among 8,177 unique expressed sequences (1.6 %). BLAST sequence comparisons between the *U. maydis *and *C. neoformans *genes identified 9 that are alternatively spliced in both species. Despite differing analytical methodologies, the estimate here of 3.6 % alternative splicing in *U. maydis *does not differ markedly from these other fungi. However, in all cases, the estimates were far less than that observed in "higher" eukaryotes (e.g. humans), where as much as 74% of genes may be subject to alternative splicing [58-61]. The low level might suggest that, among fungi, alternative splicing is of marginal biological significance. However, this interpretation ignores the importance of EST coverage when identifying alternatively spliced genes [62]. Prior to the accumulation of large numbers of ESTs, estimates of the proportion of human genes subject to alternative splicing were as low as 5 % [63]. In contrast, present estimates were based on analyses that may incorporate millions of ESTs (e.g [58]). If only the relevant subset of *U. maydis *genes were considered (those containing at least 1 intron and covered by more than 1 EST) the frequency of alternative splicing rises to > 25 %; while this is not a definitive estimate, it does support the notion that limited EST coverage may lead to an underestimate of the frequency of this phenomenon in *U. maydis*, and fungi generally.

Alternatively spliced transcripts may arise from the exclusion of an entire exon (exon skipping), the use of alternate 3' and/or 5' splice sites, or intron retention. With the exception of *C. neoformans *[17], reports of alternative splicing in fungi have been largely restricted to examples of intron retention. In contrast, the dominant form of alternate splicing in mammals is exon skipping (38 % of events) with intron retention being observed infrequently (3% of events) [59,64]. The results presented here indicate that, in *U. maydis *intron retention is the major form of alternate splicing, accounting for 59 % of the observed events (Table 3); however, all forms of alternate splicing occur. Therefore, as is the case for some fungi and mammals [17,65] there are multiple forms of alternate splicing in *U. maydis*.

Like *U. maydis *and *C. neoformans*, intron retention is the predominant form of alternate splicing in several plant species[66,67]. Wang and Brendel [68] suggest that this may be the result of differences in the mechanism of intron excision. Specifically, mammals utilize (primarily) an exon definition mechanism whereby splice sites are paired across exons. Under this model, the failure to recognize a 5' splice site results in "skipping" of the preceding exon [69]. In contrast, Wang and Brendel [68] suggest that intron definition may be the more prevalent mechanism of splice site pairing in plants. In this case, failure to recognize the splice site leads to retention of the intron. Given that fungi are thought to utilize an intron definition mechanism [70,71], it may be that of intron retention in *U. maydis*, and fungi generally is the result of a failure to recognize splice sites.

Human exons that were subject to alternative splicing tend to be bordered by splice sites that were degenerate relative to those of constitutively spliced exons [15]. In light of this observation, and given that intron retention was the principal form of alternative splicing in *U. maydis*, retained introns were compared to those that were constitutively spliced (based on available evidence). Of the 50 retained introns analyzed, 19 did not contain standard splice site dinucleotides (38 %); the corresponding proportion among the constitutively spliced introns was 5.2 %. Further, among those retained introns bearing standard splice site dinucleotides, the sequences surrounding the splice sites and branch points were more degenerate than those of the constitutively spliced introns (Figure 5). Ast [15] proposes models for the evolution of alternative splicing in metazoans that predict degeneracy of the splice sites surrounding alternatively spliced exons. Extension of these models to *U. maydis *may account for the degenerate splice sites associated with retained introns. In general these models were based in two possibilities, either intron retention is unregulated, resulting from splicing errors due to weak splice sites, or intron retention is controlled and there is a relaxation of splice site recognition that results in reduced sequence conservation. Future experiments in *U. maydis *designed to distinguish between these possibilities will include determining the proportion of alternative splicing events that are regulated.

Based primarily on the findings in humans and/or mice; organisms in which exon skipping is the principal form of alternative splicing, the canonical role of alternative splicing is to facilitate the synthesis of multiple, distinct, transcripts from a single gene. In turn, this allows for diversification of the proteome without a corresponding increase in the number of protein-coding genes [65]. In contrast, a large proportion of intron retention events result in frame shifts and this may lead to the incorporation of premature stop codons [72,73]. The high level of intron retention in *U. maydis *would not result in a substantial increase in proteome diversity. However, intron retention might serve as a mechanism for the posttranscriptional regulation of gene expression. If this were the case, it could occur either by the coupling of intron retention to mRNA degradation [73] or the regulation of intron removal, and the ensuing protein production. The latter could be linked to a developmental stage or cell type analogous to the MER2 gene of *S. cerevisiae *[74].

### Antisense transcripts

Comparing transcript and genome sequences allowed the identification of sense transcripts for 4,675 genes and antisense transcripts for 210 genes. For um02794, a gene that had multiple ESTs supporting the existence of a sense and antisense transcripts, RT-PCR using strand-specific primers for first strand synthesis detected both transcripts in haploid cells grown in two nutritional conditions. The primers used in the PCR reaction amplified sense or antisense RNA corresponding to the sense open reading frame. As such, no knowledge of the start or stop of either sense or antisense transcription was gained nor was there knowledge of the cell compartment from which each transcript was isolated. There was RT-PCR confirmation of an in silico prediction of sense and antisense transcription of a *U. maydis *gene. If this confirmation was extended to other in silico predictions of antisense transcription it would support the existence of multiple natural antisense transcripts (NATs) in *U. maydis*.

The role of the NATs in *U. maydis *is not known. They could represent an increase in genome coding potential since 181 contained potential open reading frames (Additional File 7). However, this phenomenon has not been widely noted in other organisms, this may indicate an evolutionary pressure to select against overlapping, co-transcribed genes. A possible explanation for such evolutionary bias is transcriptional interference, that is, the restricted elongation of overlapping transcripts by RNA polymerase II, as described in *S. cerevisiae *[74]. If this interference exists in *U. maydis*, separate non-overlapping ORFs would be more sterically favoured for transcription, especially with a relatively low gene density. The average intergenic distance was 973 bp in *U. maydis *[31]. With this possible inhibition and the existence of antisense transcripts that do not have potential coding capacity, a role in regulation was considered more likely. However, coding and regulation are not exclusive since transcriptional interference itself might be a gene regulatory event where the increase transcription of one ORF induces the suppression of its co-transcribed antisense counterpart.

There is evidence that antisense transcripts can regulate intron splicing in mammals [75] and there is a bias toward intron containing genes among those with antisense transcripts in *A. thaliana *suggesting a link between antisense and introns [76]. There was no such bias in the presented *U. maydis *data although some of the antisense was to intron containing genes (data not shown).

The *U. maydis *genes with antisense do not have high sense strand representation in the libraries; this could be the result of sense transcript degradation. The most studied NATs are the 21–25 nt short interfering RNAs (siRNAs) or the ~22 nt microRNAs (miRNAs) that have been found in plants, and animals [25,77]. The small RNAs are created through the action of the RNaseIII, DICER, and both can mediate cleavage of complementary RNA through a multi-protein RISC-complex [24]. SiRNA can also mediate DNA methylation leading to gene specific transcriptional silencing [26] and miRNAs can form ribonucleoprotein complexs (miRNPs) that bind to the 3' UTR of the target transcripts inhibiting translation [24]. In the fungus *N. crassa*, two mechanisms, quelling and meiotic silencing by unpaired DNA (MSUD), induce gene silencing by RNA degradation. Both processes require the action of DICER and a RISC-complex; proteins conserved among many of the fungi that exhibit RNA gene suppression [27]. However, a recent comparative genomics study showed that, *U. maydis *lacks these RNA mediated silencing components [28]; indicating that *U. maydis *cannot form miRNA or siRNA by the traditional means and, if the antisense transcripts have a roll in controlling gene expression, it must be through some other mechanism.

In a previous test for a regulatory role of antisense RNA expression, sense and antisense transcripts of *PYR3*, a dihydroorotase, were expressed in *U. maydis*. The amount of enzyme activity was unaffected by overexpression of antisense transcripts [28]. However, antisense transcripts of PYR3 are not NATs and it may be that the control in *U. maydis *is highly regulated or linked to specific genes or target sequences. *C. albicans *and *S. cerevisiae *also lack DICER and RNA dependent RNA polymerase,[78] RdRP [27], yet *C. albicans *has the ability to suppress genes with anti-sense RNA [79] and RNA complementary to the 5' UTR was shown to trigger gene silencing in *S. cerevisiae *[23].

While the role of antisense transcripts in *U. maydis *is currently unclear, the presence of RNA mediated gene silencing in *C. albicans *and *S. cerevisiae *indicates the existence of uncharacterized mechanisms that do not require DICER like proteins and these could also be functioning in *U. maydis*. Furthermore, two RNA silencing pathways were recently identified in *Drosophila melanogaster *[80], suggesting that other pathways remain to be discovered. The identified antisense transcripts in *U. maydis *provide a starting point for uncovering new roles for these molecules in cellular function.

## Conclusion

The EST analyses carried out here provide evidence that the control of nitrogen metabolism gene expression in *U. maydis *differs from the laboratory model fungi and may be similar to that of other plant pathogenic fungi. The predominance of intron retention in *U. maydis*, other fungi, and plants suggests that it may have a role in regulating gene expression. The observation of other modes of alternate splicing in *U. maydis *confirms the suggestion that this process is more diverse in fungi than previously recognized. The identification of antisense transcripts in *U. maydis *provides for the potential discovery of methods of regulating genes expression not described in eukaryotes. All of this data supports the continued development of EST resources for reasons beyond facilitating gene identification.

## Methods

### Strains and culture conditions

*U. maydis *strains 518 and 521 were originally isolated by Robin Holliday. Sally Leong, University of Wisconsin, Madison, WI, USA, has maintained a frozen stock of these cultures and the stocks used here were obtained from her in the mid 1990s. The strains have been maintained as frozen stocks at -80°C since they were obtained. When grown from frozen stocks the cultures are streaked to single colonies. They are tested for ability to cause disease at regular intervals however, they have not been single spore isolated in the classic sense. The pathogenic cycle involves the formation of diploid teliospores and when these spores germinate they undergo meiosis therefore single spore isolates obtained following infection would have a different genetic makeup. For RNA isolation from haploid cultures and growth curves, the frozen stocks were streaked on complete media plates. From these plates, single colonies of *Ustilago maydis *strain 521 were inoculated into tubes containing 3 mls of complete medium (CM, [81]) and incubated overnight at 28°C on a rotary shaker (250 rpm). For larger cell volumes 500 μl of a 3 mL overnight culture was used to inoculate 500 mls of CM in a 2 L flask and this was incubated at 28°C on a rotary shaker (250 rpm). For the construction of the CM library, cells were harvested for RNA isolation when the 500 ml culture reached an OD_600 _of ~1.2. In the case of haploids grown in minimal media, (MM [29]), MN and MC, cells were grown overnight in 500 mls of CM, note this was a short overnight and cells were harvested well before reaching stationary phase, harvested by centrifugation, washed with cold sterile double distilled water and re-suspended in 500 mls of MM, MC, or MN media respectively. The cells were allowed to grow at 28°C, on a rotary shaker at 250 rpm for an additional 12 hours before being harvested for RNA isolation. In MC and MN media the cultures did not reach an O.D. of 1.2 and RNA isolated from more than one flask of cells was used for library creation. With this approach no media is carried over and therefore there is no external source of nitrogen or carbon for the MN and MC conditions respectively. There may have been nutrients carried over in the cell walls or as internal stores in the haploid cells however, the concentration of external nitrogen and carbon created by possible efflux was not measured; therefore, absolute concentrations in the media were considered very low but not zero. For growth curves the same growth regime was used except that the optical density was determined for three 1 ml samples from each MM, MC and MN flask at the times indicated in Figure 1. Three biological replicates of this growth process were included in the growth curves reported in Figure 1. Microscopic observation of the haploid cells grown under each nutritional regimen was carried out using a Zeiss Axioskop compound microscope (Spectra Services, Webster, NY), with a QICAM digital CCD camera (QImaging, Burnaby, BC, Canada) utilizing Northern Eclipse Image Analysis software V6 (Empix Imaging, Mississauga, ON). Cells were photographed at the same growth stage as those used for RNA isolation. Growth conditions for the filamentous diploid and dormant teliospore were as described in Nugent *et al*.[29] and Zahiri *et al*[45], respectively.

### RNA isolation

Total haploid cell RNA was isolated by TRIZOL extraction following the manufacturer's suggested protocol (Invitrogen Canada, Burlington, ON, Canada). mRNA was isolated using an mRNA Purification Kit from GE Bioscience (Baie d'Urfe, Quebec, Canada) and concentrated to ~1 ug/ul, following the manufacturer's suggested protocol. RNA was isolated from filamentous diploid cultures as outlined in Nugent *et al*. [29] and dormant teliospores (TD) as described by Zahiri *et al*[45]. Teliospores were isolated from several independent infections of corn and pooled before isolation.

### Library construction

The focus of this work was genome annotation. In creating the cDNA libraries from haploid cells two methods were used to determine which captured a greater percentage of full-length cDNAs, showed the lowest redundancy and was the most efficient to use. The MC library was constructed using a modification of the CAP-Trapper method [78]. Modifications included increasing the amount of starting mRNA to 20 μg, the addition of T4gene32 protein during first strand synthesis [82], the use of the plasmid pDNR-Lib (Clontech, Mountain View, CA, USA) for cDNA cloning, and the omission of radioactive labelling to monitor first strand synthesis. The CM and MN libraries were constructed using the Primer-Extension protocol of Clontech's SMART cDNA library construction kit. For the construction of each haploid library 5 μg of mRNA was used as a template for first strand synthesis. The TD library was constructed from 1 μg of TD RNA, as per the LD-PCR protocol of the SMART cDNA library construction kit. In all cases, cloned cDNAs were transformed by electroporation into ElectroMAX™ DH5ά-E™ cells (Invitrogen) and the resulting colonies were inoculated into 96-well microtiter plates containing LB media and chloramphenicol (30 μg/ml). Microtiter plates were incubated overnight, on a rotary shaker (250 rpm) at 37°C. Glycerol was added to a concentration of 15 % (v/v) and the plates were stored at -80°C.

### DNA sequencing

Plasmid DNA was isolated from *Escherichia coli *using Whatman's 96-well mini-prep system following the manufacturer's suggested protocol (VWR International, Mississauga, ON, Canada). Nucleotide sequences corresponding to the 5' end of the mRNA were determined using the Applied Biosystems' BigDye^® ^terminator v3.1 cycle sequencing kit (Applied Biosystems, Foster City, CA, USA). Reactions were set up in 96-well plates using a master mix with each 10 μl reaction containing: 1.33 μl Ready Reaction Premix (Applied Biosystems), 1.33 μl Big Dye dilution Buffer (Applied Biosystems), 0.16 μM M13 Primer (5'-GTAAAACGACGGCCAGT- 3'), 0.2–0.3 μg template DNA, and 5.308 μl sdH_2_O. Cycling conditions were as per the manufactures suggested protocol except that they were carried out for 35 cycles. Reaction products were purified by ethanol precipitation following the manufacturers suggested protocol (Applied Biosystems), and analyzed using an ABI PRISM^® ^3100 Genetic Analyzer (Applied Biosystems).

### Sequence editing

Raw sequence data were imported into the SeqManII module of Lasergene v.5.0 (DNASTAR, Madison, WI, USA). Default settings were used to remove ambiguous bases and vector sequences. Manual editing following BLASTN [83] searches of FASTA files exported from SeqManII against the vector sequence provided by the manufacturer ensured complete removal. Given that *in planta *growth is required for teliospores production there was the possibility that *Z. mays *transcripts were present in RNA isolated from teliospores preparations. Similarly, there may be transcripts from endophytic fungi [2]. To eliminate potential contaminating sequences, ESTs in the TD and T11 libraries were compared to a *Z. mays *EST database (*Zea mays *ESTs, TIGR, Release 4.) and to the *Fusarium graminearum *genome sequence [44]. ESTs that had a better match in the non-*U. maydis *databases (i.e. lower e-values than the corresponding *U. maydis *hit) were discarded. The resulting cDNA sequences were used in BLASTN searches against the *U. maydis *genome [2]. Sequences from previously constructed germinating teliospores, T11, and filamentous diploid, D12, EST libraries were also used in these searches [29,30]. For the BLAST alignments, cutoffs of 1e-50, 1e-20, 1e-5 were created the alignments were visually inspected. Since the e-value cut-off is imposed on individual hsps (high-scoring segment pairs) and several hsps compose a complete genome match, small hsps such as those corresponding to an exon that might have been partially sequenced at the beginning or end of an EST would not be aligned to the genome accurately if the e-value is set too low (i.e. if the stringency of identifying a match was too high). The 1e-50 cut off would remove too many accurate matches. In contrast 1e-5 was not stringent enough and it was determined that a 1e-20 cut off retained accurate genome alignments without also including spurious matches. The ESTs that were not complementary to the genome (at E < 1e^-20^) were compared, using BLASTN, to the *U. maydis *mitochondrial DNA (at E < 1e^-20^) and to the rDNA region of the genome (at E < 1e^-20^). ESTs that did not hit to either of these databases were searched against the NCBI nr database using BLASTX with a cutoff e-value of 1e^-5^.

### Contig assembly

ESTs that aligned to the same region of the genome without a gap between them were combined into a contig. This means that if two ESTs overlap in the genome or align to contiguous sequences without a gap, they were considered to be in the same contig. The contigs were manually examined and those assembled based on alignment with divergent or multiple regions of the genome were eliminated from further analyses.

### Assignment of contigs to MUMDB genes

A gene identity was assigned to a contig if its genome coordinates overlapped the coordinates of a MUMDB gene. Contigs that were not assigned to a MUMDB gene were searched against the NCBI non-redundant database using BLASTX with a cut off value of 1e^-5^.

The MUMDB genes assigned to contigs composed of ESTs from all libraries (CM, MC, MN, TD, T11 and D12) were compared to a list of *S. cerevisiae *genes that were essential for growth in rich media [84]. This was accomplished by aligning the identified *U. maydis *predicted genes to the *S. cerevisiae *gene set [33] using the BLASTX with a cutoff 1e^-5 ^and identifying potential essential genes by comparison to the list. *S. cerevisiae *essential genes that aligned with a MUMDB gene were functionally categorized based on the MIPS FUNCAT system [33].

### Analysis of differences in genes represented in the EST libraries

Gene expression underlying the haploid cell response to different growth conditions was examined by functional categorization of assigned genes using the March 2006 update of the Functional Catalogue (FUNCAT)[33] system from MIPS [32]. To assess potential differential regulation contigs were ordered according to the relative number of ESTs present from each library using the method of Stekel *et al*. [34]. Each contig was assigned an *R *statistical value with the higher *R *statistical value indicating a greater degree of differential expression in a given library. While statistically well-supported data can only be obtained from this method if the libraries were created by the same method, here, this analysis was used as a screen to identify genes that were potentially differentially regulated. Reverse transcription followed by quantitative polymerase chain reaction (RT-qPCR) was used to confirm differential expression of select identified genes.

RT-qPCR was carried out by the method of Zahiri *et al*. [45] using RNA from dormant teliospores as well as from CM, MC, and MN grown haploid cells. For the analysis of genes involved in the response to nitrogen depletion, RNA from haploids grown in minimal media was also included. Isolated RNA was treated with DNaseI (Invitrogen), purified using the QIAGEN RNeasy Kit (QIAGEN, Mississsauga, ON, Canada) and concentrated by ammonium acetate/ethanol precipitation. Reverse transcription was carried out on 5 μg of DNaseI treated RNA using Superscript III (Invitrogen) and oligo (dT)_12–18 _primers (Invitrogen). The synthesized cDNA was diluted 1:50 in deionized water and 5 μl of the dilution was used as template for qPCR. The cycling conditions were 95°C for 10 mins followed by 35 cycles of 95°C for 30 sec, 60 to 62°C for 30 sec depending on the primers used (Table 5), 72°C for 1 min and a final extension of 72°C for 7 mins. The data were collected and analyzed on an ABI7700 using Sequence Detection System Version 1.7 (Applied Biosystems). Three biological replicates (with three technical replicates each) of each haploid RNA source were used in this analysis. Two biological (with three technical replicates each) were used in RT-qPCR of teliospore RNA.

**Table 5:** Primers used for RT-qPCR

Contig ID	MIPS ID	Predicted or Known Function	Outside primers (5' to 3')	Inside primers (5' to 3')
Contig 1438	--	--	F: CGAGCCAAGTGGAGGAAGAAGCGTA	F: TGCGCGCCCGATCCAAGTAAAC
			R: ACGCTTTAATTTTTTTGGGGCATGTTCT	R: CGCATAGACAAGCAACGACGGTAGGA
Contig 4572	UM01426	Conserved Hypothetical Protein	F: TCCGACTGCAATCTCTACCCGAAACTT	F: GCGCTCGTCAACACAATCATCCATG
			R: AGGCGAACAGCGGTGGAATCAAC	R: GGGCAAAAGTATCAAGACCAGCATCAGAG
Contig 4070	UM00919	Probably ADP, ATP Carrier Protein	F: CGTCTCGCCGCCCCCTACAA	F: TTCCCTCGGTTGTCGGTATCGTTGT
			R: CGGCACCCTTGAAGAGCGACG	R: ACACCCTCGGCGGCAATGATG
Contig 1652	UM04481	Related to Alcohol Dehydrogenase II	F: GCTGCCACCATCAAGGACTTCAAAAA	F: CCACCTCAAGCCCGACAAAACATTC
			R: GACCCTGCAGCTTCTTCGAGTTGTAGTG	R: TCGAAGGGTCCGTCAGCTGGTAAAC
Contig 2167	UM04974	Putative Protein	F: CGCCTGCTGCTGAATCGGACC	F: TCGGGCTGGAGCGTTGATCTGA
			R: CGGACATGGACAAAGACTCGACGTG	R: GCGCGGTTTGGTCGACATCGT
Contig 1015	UM11105	Nitrate Transporter	F: CGCTCTGCTCATTCTCGGTGCTATTC	F: CGCACTGCCGACAACGAAAAGG
			R: GGGTGGGTGAGCGGTTGCAGTAA	R: GCGGCGTCGATAGCGGTGAAA
Contig 4494	UM01756	Probable Purine Transporter	F: CGGCTCGCTCGTCGGTTGCT	F: CCCCGCCTTTGTCACCATCGTC
			R: CGCTCCTCTCGTTCTCCAGTTGCTC	R: CCGCGGGAGAGCTTGAGGATGAT
Contig 992	UM03847	Nitrate Reductase	F: TCGTCGTCATCACCGGCATCCTC	F: GCAAGCCGTTCCTCGATCCCAAG
			R: TGGCTTCGTTTTCAGGAGTGCAATTG	R: TCTTTGTACCATCTCTGTCTCGCCTGTTCT
Contig 1755	UM04577	Probable Urea Permease	F: GAAGGGGCTGGCTGGGCTGTAGT	F: TGCTTACGCGCGCCATCAATG
			R: GGCAAGACACCAACAGCGAGCG	R: GCGTCGAGTCCGGCAGCTTTG
Contig 3380	UM06012	General Amino Acid Permease	F: GAAGCCAAGAACCCGCGCAAGT	F: TACCAACTACATCCCGATCCCCTTCTTC
			R: GTCGTGGGTTTCTCGTCTGGCTGTT	R: GTCGTGGGTTTCTCGTCTGGCTGT
Contig 3396	UM06045	Probable Urease	F: ACTCCACCATGCCGGCTCCCTAG	F: CTCCATCCCCACAGTGCAGCCTATC
			R: GCGTCAATGGCAACTCGTCAGCTG	R: CCCCTTGACCCCCTCGATTCTCTT
Contig 3235	UM05889	Ammonium Transporter UMP2	F: GGCACGCCCACCGAACTCATCTAT	F: TCGACATCTTTGCTGCTCACGGTATTG
			R: TCGGTGGATGCAGTAGCGTGATGAA	R: TGGCGAACAGCAGGATGAGCGTAA

### Identification of full-length cDNAs

cDNAs potentially containing full-length reverse transcripts were identified by one of three methods. 1) For clones represented by ESTs that were assigned a MUMDB gene, cDNAs were classified as potential full-length if the EST 5' end aligned to or was upstream of the translational start predicted by MIPS. 2) For clones represented by ESTs that were not assigned a MUMDB gene, the EST was searched against the NCBI non-redundant database (NR) with the BLASTX algorithm and a 1e^-5 ^cut off. The ESTs and BLASTX results were submitted to the web based program TargetIdentifier [85] that predicted which ESTs represented full-length cDNAs. 3) For cDNA clones represented by ESTs that were not assigned a MUMDB gene and did not have a significant match in the NR, the ESTs were submitted to the web based program OrfPredictor [86] and those that contained a predicted open reading frame that starts with a methionine (AUG codon) were considered full-length.

### Intron analysis and identification of alternative splicing

GeneSeqer [87] was used to generate putative transcript structures based on an alignment of ESTs to the corresponding genomic loci. Default parameters were used with the following exceptions: the MinMatchLen was 30, and both the MinQualityHSP and MinQualityCHAIN were set to 45. A minimum exon length of 5 nts was chosen and a minimum quality score of less than 0.9 was applied to exons. A *Schizosaccharomyces pombe *splice site model was used and the join length was set to 0 nts. On the basis of these alignments, GeneSeqer also generates gene structures (i.e. intron/exon positions) for each of the PGLs. The occurrence of more than one predicted gene structure for a given PGL may indicate a possible alternate splicing (AS) event. Because the PGLs are assembled from cDNA sequences using the genome sequence as a backbone AS events can be distinguished from alignments formed by any suspected chimaeric cDNA clones. If the alignment included disparate regions of chromosome (i.e. cDNAs assembled from distinct non-adjacent genes) these would not be called by the software and the cDNA sequence producing this result would not be included in succeeding analyses. If the PGL spanned a large region, for example linking adjacent genes on the chromosome these would also be removed by the software or at least flagged and then they would have been removed by visual inspection. For every alternate splicing event identified by the software the alignments of the cDNA and genomic sequence were visually inspected to ensure accuracy.

The results generated by GeneSeqer were parsed using a collection of Perl scripts. A Microsoft Access database was populated with the genomic location of each putative transcript structure and the corresponding intron and exon coordinates. Genomic locations with two or more predicted transcript structures were flagged as being subject to alternate splicing. The EST/genome alignment at each of these loci was examined using MyGV 1.0 [35] to identify and remove transcript structures resulting from low quality sequences or misalignment.

The remaining loci were categorized based on the nature of the alternative splicing event and the amount of EST support. Categorization was carried out as follows: For each PGL, a reference transcript structure was selected. The remaining structures were classified relative to this reference. When multiple exons were skipped (or multiple introns retained) in a single structure it was treated as only a single occurrence of the event. When two (or more) distinct forms of alternative splicing were apparent in a single structure (e.g. exon skipping and intron retention) it was included in both categories. The categories were: 1) Single EST IR – Intron retention was implied by only a single EST, 2) Multiple EST IR – Intron retention was implied by multiple ESTs, 3) Processed IR – An intron was retained in a transcript structure that also showed successful splicing of another intron, 4) Alternative donor site selection, 5) Alternative acceptor site Selection, and 6) Exon Skipping.

To facilitate ready comparison to other fungi, the intron dataset was analysed, using components of the FELINES tool kit [88], in a manner similar to that of Kupfer *et al*. [57]. Briefly, the construction and analysis of each of the intron datasets was carried as follows: A Perl script was written to extract those regions of the genome sequence corresponding to the introns identified by GeneSeqer. The sequences were compiled in a FASTA-formatted file and filtered to remove those lacking acceptable splice site dinucleotides (i.e. the two nucleotides at the beginning and end of each intron) and those less than 20 nts. The acceptable splice site borders were: GU/AG, GC/AG, AU/AC, AU/AG, and AU/AA. The reason for the removal of short introns is that the 5'/3' splice site of a 20 nt intron would be the same. The filtered intron datasets were analyzed using the perl script "icat" from the FELINES toolkit [88]. This identified potential branch points in each intron and generated alignments of these and the 5'/3' splice sites. Weblogo was used to produce sequence logos based on these alignments.

### RT-PCR assessment of alternate splicing

Transcript structures of select genes, identified as being subject to alternate splicing, were investigated using reverse transcriptase-PCR (RT-PCR). RNA isolated from dormant teliospores, filamentous diploid cultures and haploid cells grown in CM, MC, or MN media, was used to prepared cDNA as outlined for the RT-qPCR analysis. cDNA from two biological replicates for each growth condition/cell type was used in 50μl PCR reactions as outlined for the antisense transcript analysis except that the primers used were those outlined in Table 6. The cycling conditions were: 95°C for 10 minutes followed by 30 cycles of 95°C for 30 sec, 67 to 62°C (depending on the primers used) for 30 sec, 72°C for 1 to 3 mins (depending on the product length expect) and a final extension of 72°C for 7 minutes. PCR products were analyzed by agarose gel electrophoresis.

**Table 6:** Primers for RT-PCR confirmation of Alternate Splicing

**Predicted Gene Locations**	**Primers (5' to 3')**	**Annealing temperature (**°**C)**
1.168_PGL_17 um04632	F: CACGACACTTTTCTCAAAGTACCTA	57
	R: CGTCATCGGACTCTTCCTTC	
1.129_PGL_4 um10506/um10507	F: ATATACCATCGGGACCGTCA	57
	R: ACCCATCGAGGAGAGGAAGT	
1.139_PGL_18 um11744	F: CCATCCTTCACTCACACACG	58
	R: GCTGCAAAATACGCGTACAG	
1.84_PGL_9 um02514s	F: TCACTTTGCTCGATGCAGTT	58
	R: GGATCCGACTCGATTGAAGA	

### Anti-sense transcript analysis

Potential anti-sense transcripts were identified by examining contigs that contained the anti-sense strand of a MUMDB gene. The anti-sense contigs where further investigated if they contained more than one EST and there was a sense contig supporting the MIPS gene prediction. In these instances ESTs representing both strands were aligned to the genomic sequence and to the sequence of the MUMDB ORFs using NCBI's Spidey [37]. The alignments were confirmed by visual inspection and potential ORFs were identified using ORFpredictor[86] and ORFfinder [89]. RT-PCR was carried out to assess transcript presence for a predicted gene represented by anti sense ESTs in MN library and sense ESTs in CM library. Four μg of RNA from haploids grown in CM and MN was DNaseI treated as indicated above for RT-qPCR. Four separate first strand synthesis reactions were carried out using 1 μg of DNaseI treated RNA, SuperScript III reverse transcriptase (Invitrogen) and one of the following: 1) a sense strand specific primer (5' GCTTGCTGCCTTGACCATCTG 3'; Sigma Genosys, Oakville ON), 2) an anti-sense strand specific primer (5' TCACCTCTTGCCCTTGTACACT 3'; Sigma Genosys), 3) Oligo-dT_(12–18) _(Invitrogen) or 4) no primer. Following RNaseH (Invitrogen) treatment for 20 mins at 37°C, one tenth of each cDNA was used in a 50 μl PCR reaction that included 1× Amplitaq Gold Buffer (Applied Biosystems), 2.5 mM MgCl_2 _(Applied Biosystems), 200 μM of each dNTP (Applied Biosystems), 200 nM of forward primer (5' CGTCGTTTTGCAGCCAGCCAC 3'; Sigma Genosys), 200 nM of reverse primer (5' GCTCTAATCGTGAATCGTGG AAT 3'; Sigma Genosys), and 1.25 U of Amplitaq Gold (Applied Biosystems). The cycling conditions were 95°C for 10 mins followed by 35 cycles of 95°C for 30 sec, 60°C for 30 sec, 72°C for 1 min and a final extension of 72°C for 7 mins using an ABI 9700 thermal cycler (Applied Biosystems).

## Competing interests

the author(s) declares that there are no competing interests.

## Authors' contributions

EH created the haploid cell EST libraries, carried out the functional annotation, antisense analysis as well as RT-PCR and RT-qPCR confirmations. He also wrote initial drafts for relevant portions of the manuscript. MC created the dormant teliospores library, carried out the intron analysis and wrote initial drafts of relevant portions of the manuscript. BJS conceived of the project, directed library construction, analysis and PCR confirmation experiments, as well as significantly edited the final version of the manuscript. All authors read and approved the final manuscript.

## Supplementary Material

Additional File 1 Table of genes found in all six *U. maydis *EST libraries analyzed. The table lists the MUDB annotations as well as the potential *S. cerevisiae *counterpart as determined by reciprocal BLAST analysis. Where no *S. cerevisiae *counterpart was indicated none was detected. Click here for file

Additional file 2 Table FUNCAT category and MUMDB gene assignment to ESTs. For the contigs that have a FUNCAT designation, this table lists the FUNCAT assignments and MUMDB gene assignments as well as the number of ESTs found in each library. Click here for file

Additional File 3 RT-qPCR results assessing differential gene expression predicted by *R *statistical analysis. The relative abundance of transcript for each gene was noted in the graph below the gene name. a) Genes selected only to assess differential transcript abundance predicted by the *R *statistic in TD, CM, MC and MN; b) Genes selected to assess the validity of the *R *statistics as well as to investigate the transcript abundance of genes involved in nitrogen metabolism in TD, CM, MC, MN and MM. Click here for file

Additional file 4 Table of predicted gene locations and corresponding MUMDB ORFs with evidence of alternate splicing. Alternative splicing categories were as follows: 1) Single EST IR – Intron retention was implied by only a single EST, 2) Multiple EST IR – Intron retention was implied by multiple ESTs, 3) Processed IR – An intron was retained in a transcript structure that also showed successful splicing of another intron, 4) Alternate donor site, 5) Alternate acceptor site and 6) Exon Skipping. Note: 1 "-" indicates a PGL for which unambiguous categorization was not possible. Click here for file

Additional File 5 RT-PCR amplicons identifying multiple transcript isoforms. a) Ethidium bromide stained agarose gel showing amplicons from cDNA reverse transcribed from total RNA isolated from two biological replicates for each culture condition. RNA was isolated from haploid cells grown in complete (CM), minus carbon (MC), or minus nitrogen (MN) media as well as from dormant teliospores (TD) and diploid cell cultures (DIP). The lane labelled gD represents amplification from genomic DNA. H_2_O indicates the negative control. The ladders in the outside lanes were Full Ranger (Norgen Biotek, St. Catherines, Canada; left10 μl, right 5 g μl). b) Diagrammatic representation of transcript structures for the *U. maydis *genes indicated on the left side of the figure. The lines indicate introns that are remove in the mature transcript and the boxes exons that are retained in the mature transcript. gDNA indicates the genomic DNA sequence and transcript structures are numbered on the left. The positions of primers used for PCR amplification are indicated by arrows. Click here for file

Additional File 6 Frequency histogram of EST supported *U. maydis *intron lengths. This is a figure of histograms representing frequencies of introns of different length classes. Click here for file

Additional File 7 Table of genes represented in the EST libraries as antisense trancripts. The table indicates the MUMDB predicted genes for which antisense transcripts were detected, whether there was a possible open reading frame in these antisense transcripts and whether there was a corresponding sense transcript detected in any of the six EST libraries analyzed. Click here for file
