# Supplementary figures and images for: Gene discovery and transcript analyses in the corn smut pathogen Ustilago maydis: expressed sequence tag and genome sequence comparison
Source: BMC Genomics. 2007 Sep 24;8:334. doi: 10.1186/1471-2164-8-334 (PMC2219887; doi:10.1186/1471-2164-8-334)

A

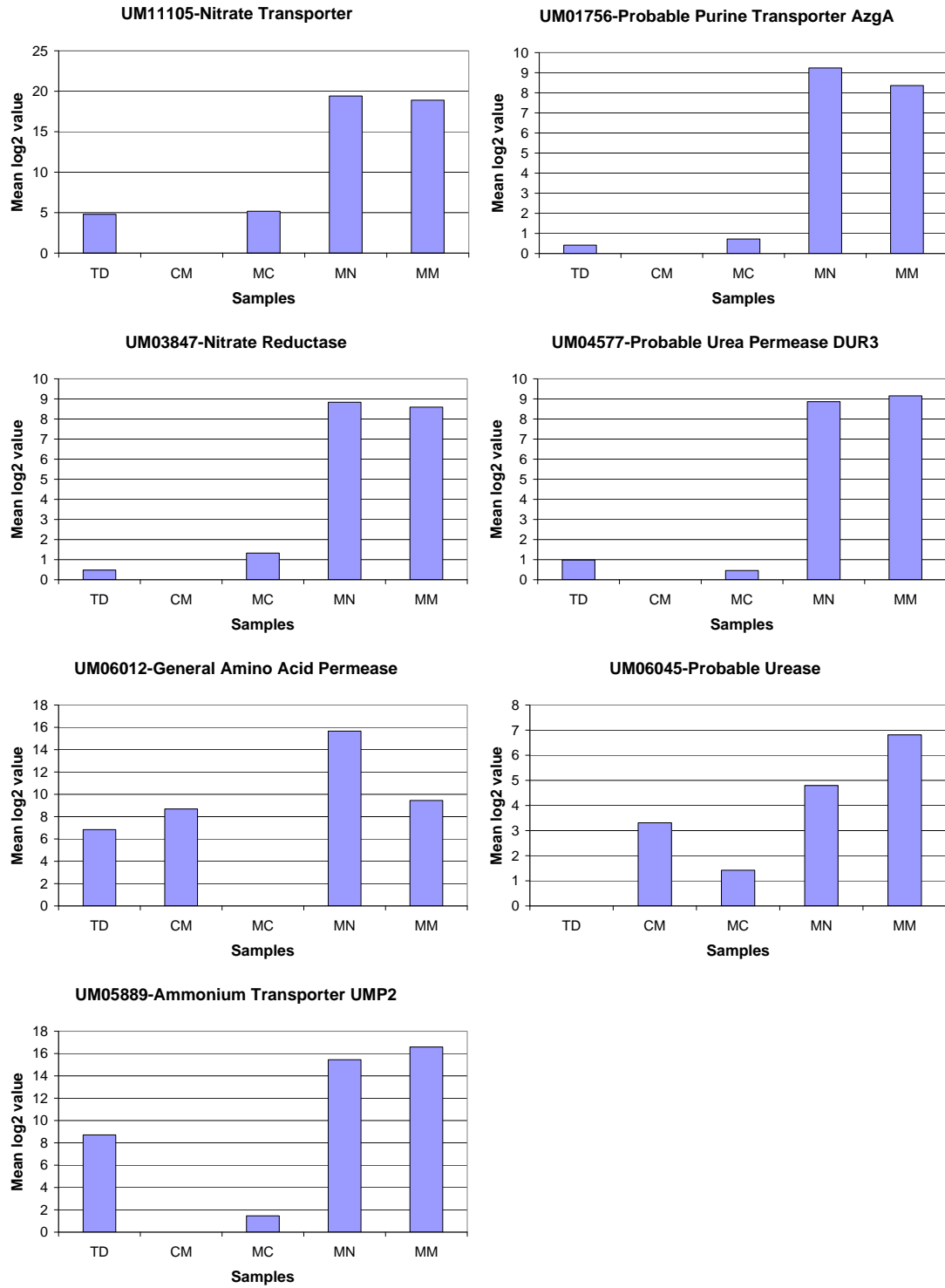

**B**

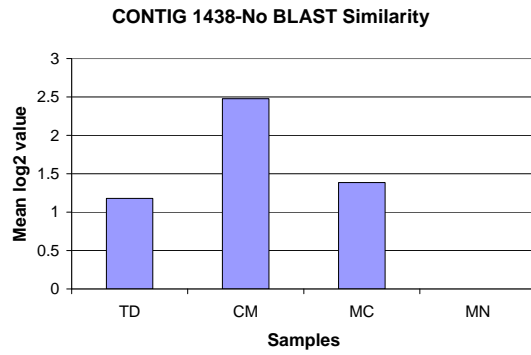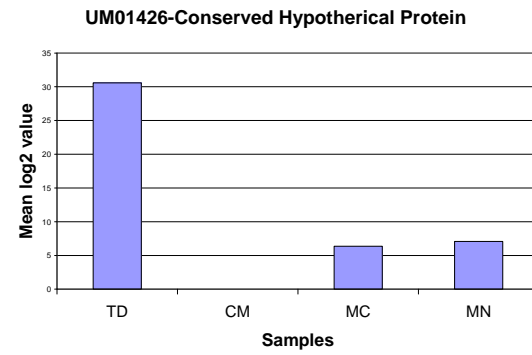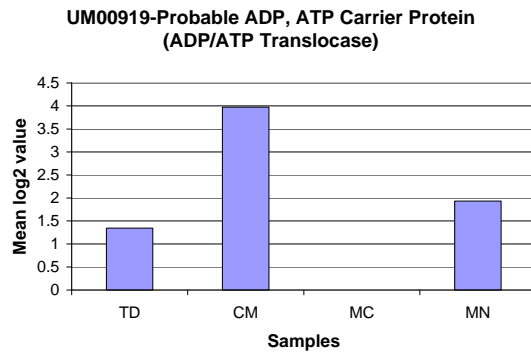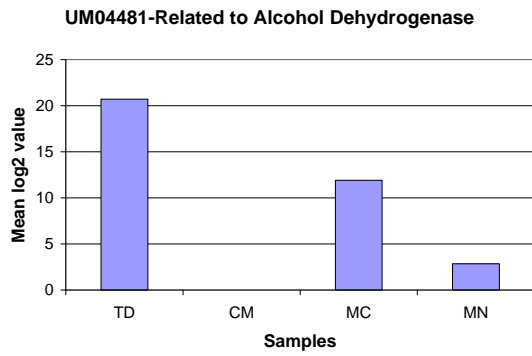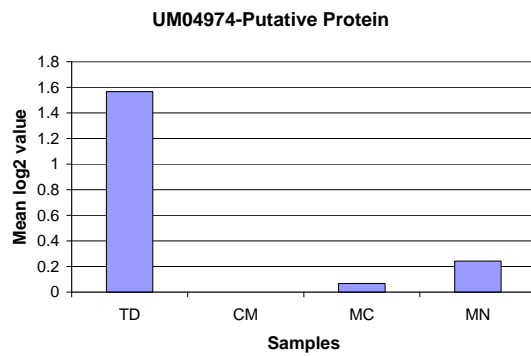

Supplement: Additional File 3 — RT-qPCR results assessing differential gene expression predicted by R statistical analysis. The relative abundance of transcript for each gene was noted in the graph below the gene name. a) Genes selected only to assess differential transcript abundance predicted by the R statistic in TD, CM, MC and MN; b) Genes selected to assess the validity of the R statistics as well as to investigate the transcript abundance of genes involved in nitrogen metabolism in TD, CM, MC, MN and MM. [file 1471-2164-8-334-S3.pdf]

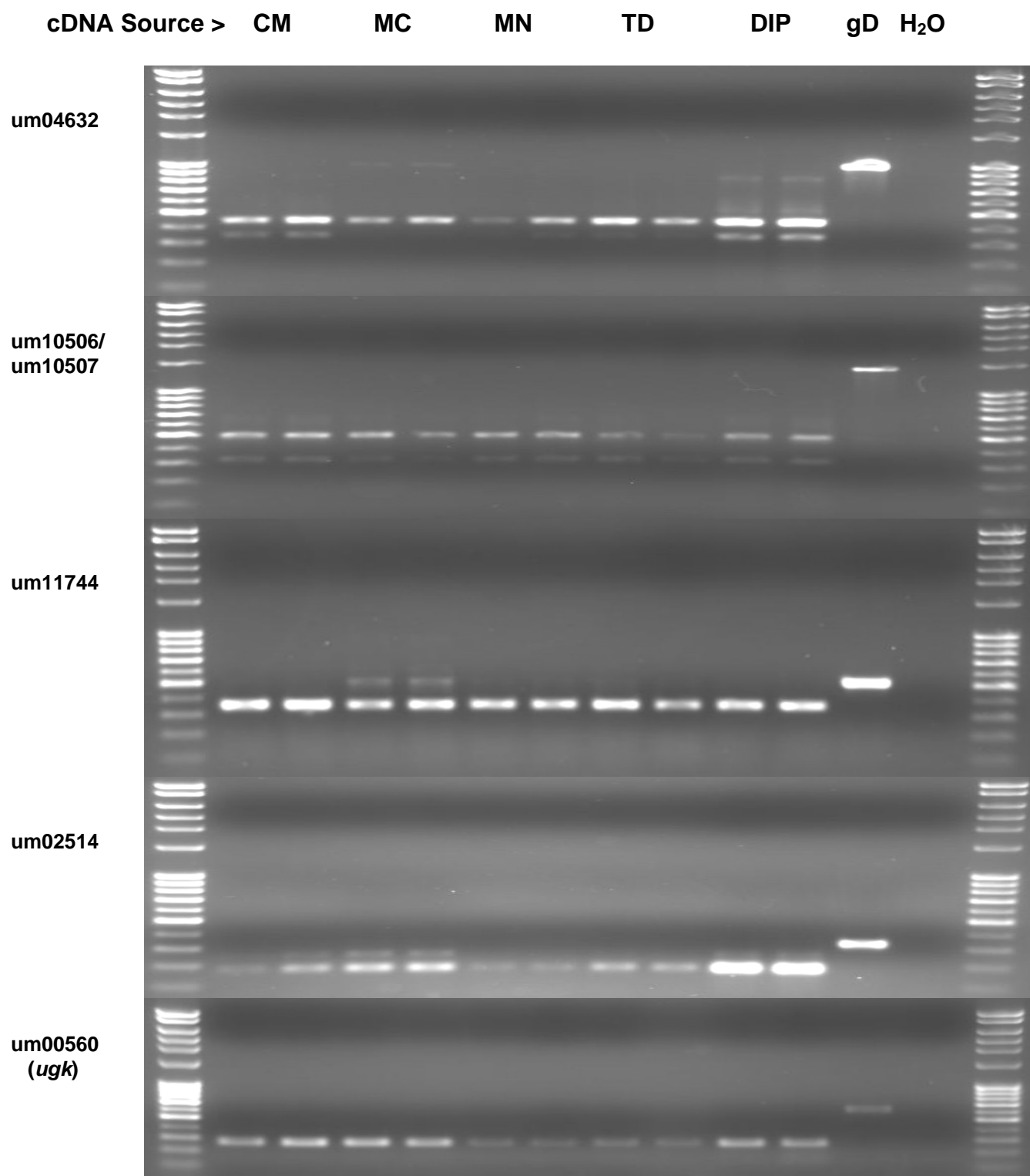

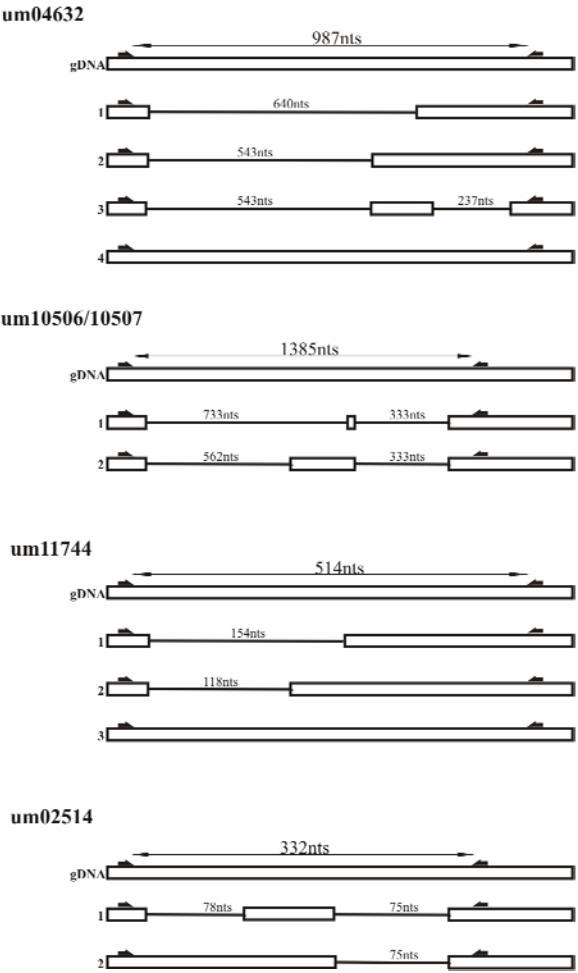

Figure 6b

Supplement: Additional File 5 — RT-PCR amplicons identifying multiple transcript isoforms. a) Ethidium bromide stained agarose gel showing amplicons from cDNA reverse transcribed from total RNA isolated from two biological replicates for each culture condition. RNA was isolated from haploid cells grown in complete (CM), minus carbon (MC), or minus nitrogen (MN) media as well as from dormant teliospores (TD) and diploid cell cultures (DIP). The lane labelled gD represents amplification from genomic DNA. H2O indicates the negative control. The ladders in the outside lanes were Full Ranger (Norgen Biotek, St. Catherines, Canada; left10 μl, right 5 g μl). b) Diagrammatic representation of transcript structures for the U. maydis genes indicated on the left side of the figure. The lines indicate introns that are remove in the mature transcript and the boxes exons that are retained in the mature transcript. gDNA indicates the genomic DNA sequence and transcript structures are numbered on the left. The positions of primers used for PCR amplification are indicated by arrows. [file 1471-2164-8-334-S5.pdf]
