# Supplementary material for: Gene discovery and transcript analyses in the corn smut pathogen Ustilago maydis: expressed sequence tag and genome sequence comparison
Source: BMC Genomics. 2007 Sep 24;8:334. doi: 10.1186/1471-2164-8-334 (PMC2219887; doi:10.1186/1471-2164-8-334)
Supplement: Additional File 6 — Frequency histogram of EST supported U. maydis intron lengths. This is a figure of histograms representing frequencies of introns of different length classes. [file 1471-2164-8-334-S6.doc]

Supplementary Figure 1: Frequency histogram of *U. maydis* intron lengths.
